# Automating Areas of Interest Analysis in Mobile Eye Tracking Experiments based on Machine Learning

**DOI:** 10.16910/jemr.11.6.6

**Published:** 2018-12-10

**Authors:** Julian Wolf, Stephan Hess, David Bachmann, Quentin Lohmeyer, Mirko Meboldt

**Affiliations:** ETH Zürich, Switzerland

**Keywords:** mobile eye tracking, areas of interest, machine learning, mask R-CNN, object detection, gaze mapping, tangible objects, cGOM, usability

## Abstract

For an in-depth, AOI-based analysis of mobile eye tracking data, a preceding gaze assign-ment step is inevitable. Current solutions such as manual gaze mapping or marker-based approaches are tedious and not suitable for applications manipulating tangible objects. This makes mobile eye tracking studies with several hours of recording difficult to analyse quan-titatively. We introduce a new machine learning-based algorithm, the computational Gaze-Object Mapping (cGOM), that automatically maps gaze data onto respective AOIs. cGOM extends state-of-the-art object detection and segmentation by mask R-CNN with a gaze mapping feature. The new algorithm’s performance is validated against a manual fixation-by-fixation mapping, which is considered as ground truth, in terms of true positive rate (TPR), true negative rate (TNR) and efficiency. Using only 72 training images with 264 labelled object representations, cGOM is able to reach a TPR of approx. 80% and a TNR of 85% compared to the manual mapping. The break-even point is reached at 2 hours of eye tracking recording for the total procedure, respectively 1 hour considering human working time only. Together with a real-time capability of the mapping process after completed train-ing, even hours of eye tracking recording can be evaluated efficiently. *(Code and video examples have been made available at: https://gitlab.ethz.ch/pdz/cgom.git)*

## Introduction

Areas of Interest (AOIs) are widely used for stimuli-driven, quantitative analysis of eye tracking data and allow the determination of important metrics such as dwell time or transitions (1). Despite the progress in eye tracking software over the last years, AOI analysis for mobile eye trackers is still an error-prone and time-consuming manual task. In particular, this applies to studies in which the participants move around and interact with tangible objects. This is often the case for usability testing in real-world applications (2). As a result of these challenges, many scientists are hesitating to use mobile eye tracking in their research even though it is often the appropriate tool for the study design (3).

Various methods exist for assigning gaze data to respective AOIs such as manual frame-by-frame or fixation-by-fixation analysis and dynamic AOIs using either key frames or different types of markers. Ooms et al. (4) state that dynamic AOIs based on interpolation between key frames are generally not suitable for interactive eye tracking studies. Vansteenkiste et al. (3) add that for experiments in natural settings, it is almost inevitable to manually assign the gaze point frame-by-frame to a static reference image or, as proposed in their paper and which is state of the art by now, using a fixation-by-fixation algorithm. These manual methods are very effective and applicable to any possible case, but also highly tedious. Over the last few years, marker-based approaches using visible, infrared or natural markers have become more and more common and are now widely used for automated computing of AOIs (5, 6, 7). Although the use of markers can accelerate the evaluation process enormously, they are limited to the types of scenes that can be analyzed (8). Applied to interactive experiments with tangible objects, they represent a potential disturbance factor for analyzing natural attentional distribution, cannot be attached to small objects due to the necessary minimum detectable size, must face the front camera for detection and generally cannot be used for objects that move and rotate during the experiment (e.g. rolling ball).

**Figure 1:**
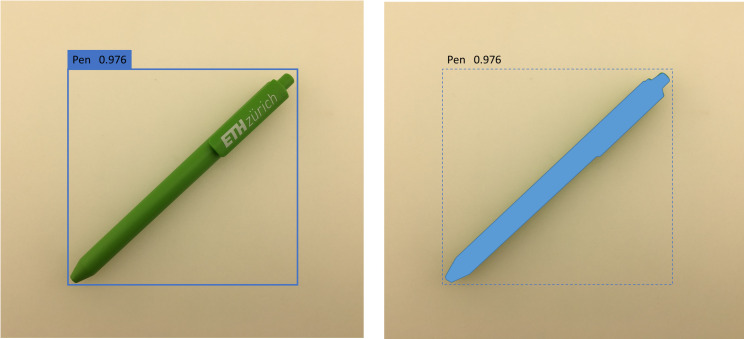
Bounding box created by a conventional deep CNN (left) and close contour mask created by mask R-CNN (right).

To overcome these limitations, object detection algorithms could be applied directly to the objects of interest and not to markers (9). In recent years, major breakthroughs in object detection have been achieved by machine learning approaches based on deep convolutional neuronal networks (deep CNNs) (10). Until recently, CNN-based object detection algorithms were solely able to roughly predict the position of an object by means of bounding boxes (11). Figure 1 (left) shows the disadvantage of such a rectangular AOI using a simple diagonally placed pen as an example. The oversize and shape of the AOI can lead to high error rates, in particular in experimental setups in which overlapping is expected (12).

In 2017, mask R-CNN was introduced (13) as one of the first deep CNNs that not only detects the objects, but also outputs binary masks that cover the objects close contour (Figure 1, right). In this article, a study is conducted to compare AOI analysis using Semantic Gaze Mapping (SGM), which is integrated in SMI BeGaze 3.6 (Senso Motoric Instruments, Teltow, Germany) and is considered as ground truth, with an AOI algorithm based on mask R-CNN being introduced here for the first time.

*Semantic Gaze Mapping.* SGM is a manual fixation-by-fixation analysis method used to connect the gaze point of each fixation to the underlying AOI in a static reference view (3). Successively for each fixation of the eye tracking recording, the fixation’s middle frame is shown to the analyst (e.g. for a fixation consisting of seven frames only the fourth frame is displayed). The analyst then evaluates the position of the gaze point in the frame and clicks on the corresponding AOI in the reference image.

*Computational Gaze-Object Mapping (cGOM)*. cGOM is based on a loop function that iterates through all fixations’ middle frames and always performs the same routine of (i) object detection using mask R-CNN and (ii) comparison of object and gaze coordinates. In detail, each frame consists of a number of pixels that can be precisely described by x and y coordinates in the two-dimensional plane with the origin in the top left corner of the image. Mask R-CNN uses plain video frames as input and outputs the frame with a suggested set of corresponding pixels for each object of interest. If the gaze coordinate matches with the coordinate of an object of interest, cGOM automatically assigns the gaze to the respective AOI.

The performance of the two evaluation methods is compared in terms of conformance with the ground truth and efficiency, expressed by the two research questions *RQ1* and *RQ2*. The goal of the study is to investigate whether the new algorithm offers the potential of replacing conventional, manual evaluation for study designs with tangible objects. Mask R-CNN, which is the core element of the cGOM algorithm, has already surpassed other state-of-the-art networks in object detection and segmentation tasks when trained on huge online data sets (13). However, since the creation of such data sets is very time-consuming and not feasible for common studies, a small and more realistically sized training data set will be used for the investigations in this article.

(RQ1) How effective is cGOM in assigning fixations to respective AOIs in comparison with the ground truth?

(RQ2) At which recording duration does the efficiency of the computer-based evaluation exceed that of the manual evaluation?

## Methods

The study presented in this article consisted of two parts. Firstly, an observation of a handling task was performed for creating a homogeneous data set in a fully controlled test environment. Secondly, the main study was conducted by analysing the data sets of the handling task and varying the evaluation method in the two factor levels *SGM (Semantic Gaze Mapping)* and *cGOM (computational Gaze-Object Mapping)*.

### Handling Task

*Participants.* 10 participants (9 males and 1 female, average 26.6 years, range 21-30 years) conducted the handling task wearing the eye tracking glasses. All participants had normal or corrected to normal vision and were either mechanical engineering students or PhD students.

*Material.* The data was collected using the eye tracking glasses SMI ETG 2 with a scene resolution of 1280 x 960 px (viewing angle: 60° horizontal, 46° vertical) of the front camera offering a sampling frequency of 24 Hz with the gaze point measurement having an accuracy of 0.5° over all distances.

**Figure 2:**
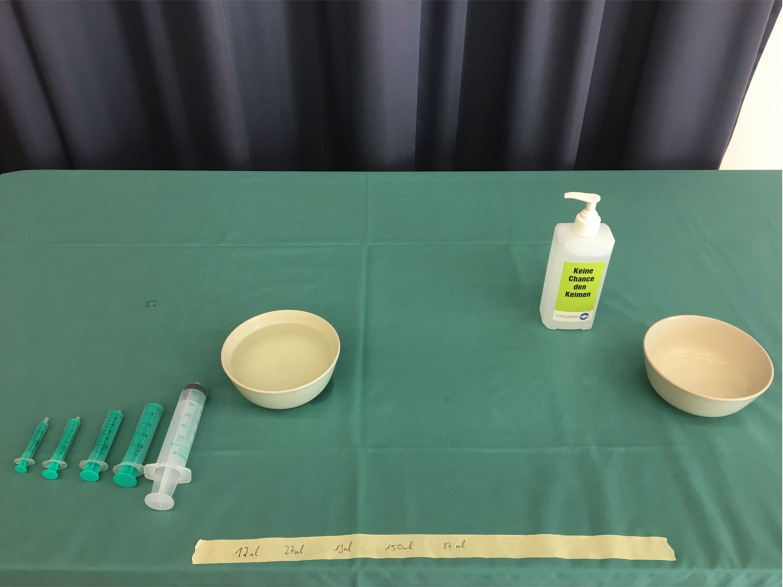
Spatial arrangement of the stimuli at the beginning of the handling task.

*Stimuli.* The stimuli (Figure 2) were placed on a table covered in a green tablecloth and consisted of five transparent syringes, two beige bowls and one disinfectant dispenser. Four of the syringes had a green piston and maximum filling capacities of 2, 5, 10 and 20 ml and one was fully transparent with a filling capacity of 50 ml.

The bowl on the left side was almost completely filled with water and the other one was empty. Moreover, there was one long piece of adhesive tape attached to the tablecloth with five filling quantities written on it (12ml, 27ml, 19ml, 150ml and 87ml).

*Task.* The participants were asked to decant the filling quantities specified on the adhesive tape from the left bowl to the right bowl. The two bowls and the disinfectant dispenser should not be moved and the participants were only allowed to use the maximum filling quantity for each syringe. After each completed decanting of one of the five preset values, the participants were instructed to disinfect their hands.

### Design

For the main study, the data set of the handling task was analyzed by the two evaluation methods *SGM* and *cGOM*. Both evaluation methods were compared in terms of conformance with the ground truth and efficiency, quantified through the two dependent variables (i) fixation-count per AOI and (ii) required time for each evaluation step. For calculation of the *fixation-count per AOI*, three AOIs were defined. All syringes were equally labelled as *syringe* without further differentiation. The disinfectant dispenser was referred to as *bottle*. All gaze points that did not affect either AOI were to be assigned to the *background (“BG”)*. True positive rates (TPR) and true negative rates (TNR) were calculated for the AOIs *syringe* and *bottle* to evaluate the effectivity of the algorithm. While TPR describes how many of the true positive assignments of the ground truth were found by the *cGOM* tool, TNR describes the same comparison for the true negative assignments or non-assignments.

Even though *cGOM* is able to assign the gaze point of each frame to the corresponding AOI, for reasons of comparability the assignment was also performed using only the fixations’ middle frame. For comparison of efficiency, the *required time for each evaluation step* of *SGM* and *cGOM* was measured and summed up. For all manual work steps, the times were averaged over all analysts, whereas all computational steps were measured in one representative run. Finally, the relation of data size and required time for evaluation was plotted and extended by a linear trend line to allow the determination and visualization of the break-even point of both evaluation methods.

### Participants & Materials

Five professional analysts (5 males, average 29 years, range 26-37 years), experienced in eye tracking data analysis, performed both the evaluation using *Semantic Gaze Mapping* and the manual steps of *computational Gaze-Object Mapping* (e.g. data labelling). For the latter, they received a training prior to execution. All operations concerning mask R-CNN were performed using an AWS GPU 1 NVIDEA Tesla V100 (Graphics Processing Unit) via Amazon Web Services cloud computing. Both Semantic Gaze Mapping and the export of gaze data were performed using SMI’s BeGaze 3.6.

### Procedure

The evaluation process is divided into three purely manual steps for *SGM* and five steps for *cGOM* with the latter consisting of two computational operations and three that demand manual execution by the analyst. The respective steps of both methods are explained in the following.

*Semantic Gaze Mapping.* Initially, the evaluation was prepared once by loading a reference image with all objects of interest into the software and drawing AOIs accordingly. This reference image and all respective AOIs can be reused over all trials. Subsequently, the manual mapping for the recordings of the handling tasks was performed for all fixations until at last, the data was exported.

*Computational Gaze-Object Mapping.* First, training images were collected to train mask R-CNN on the handling task setup, using only 72 frames from the recording of a pilot study. All images were taken from the front camera of the eye tracking glasses, resulting in a corresponding resolution of the training and the test images. Due to the small amount of training images, it was of great importance to keep environmental conditions constant throughout all studies. Mask R-CNN needs labelled images as input for training, just as they should be outputted later. To do this, close contour masks were manually drawn on all objects of interest in the training images. Once all images were labelled, the training of the neural network was started. This operation is purely computer-based and thus did not require the analyst's working time, allowing the analyst to export the gaze data in the meantime. The *cGOM* algorithm requires start time, duration and end time of all fixations, the x and y coordinates of the gaze point as well as the raw video recording of the front camera. Once all data was prepared, in a final step, the algorithm performed the gaze-object mapping of all trials in a single run.

## Results

**Figure 3:**
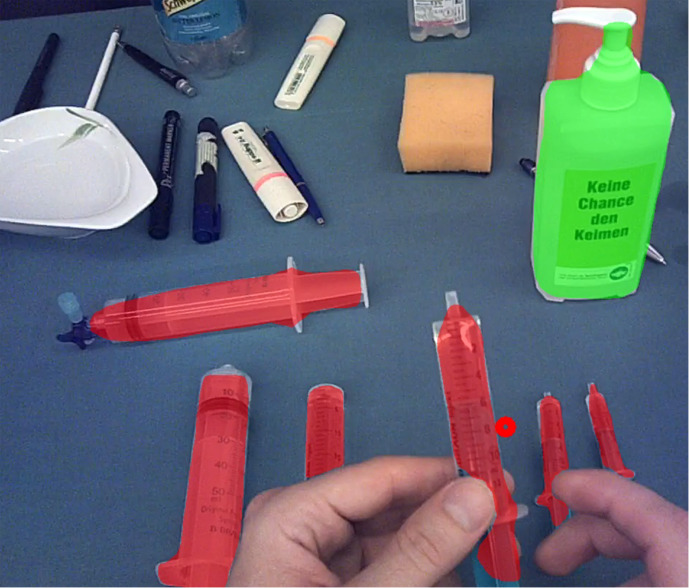
Detection of the two objects syringe and bottle by the cGOM algorithm. The detected objects are marked with masks, coloured according to the object class. Their positions in the image are then compared with the corresponding gaze point coordinate (red ring).

As presented in *Methods*, the algorithm in its core is an already established state-of-the-art convolutional neuronal network for object detection including the prediction of masks. Figure 3 shows that the algorithm is able to perform the mapping of specific gaze points to gazed-at objects when comparing the position of the object masks with the gaze point coordinates. On the one hand, this chapter shall evaluate the mapping performance of the algorithm in comparison to the manual mapping, which is considered as ground truth. On the other hand, it shall provide an overview of the time needed to operate the algorithm and present the break-even point from which on the algorithm approach is faster than the manual mapping.

**Figure 4:**
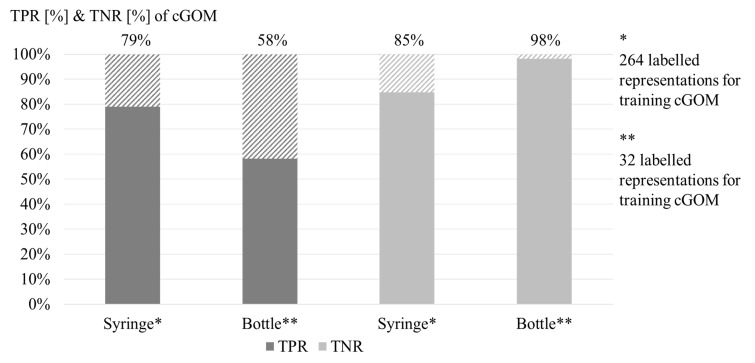
True positive rate (TPR) and true negative rate (TNR) of the cGOM assignment, compared to the manual assignment, which is considered as ground truth [%]. Relation between the results of the manual mapping (abscissa) and the mapping by the cGOM tool (ordinate). 264 labelled representations for the AOI syringe, and 32 labelled representations for the AOI bottle were used for training the cGOM algorithm.

*Effectivity evaluation.* Figure 4 shows the results for TPR and TNR of the two AOIs *syringe* and *bottle.* For the AOI *syringe,* the *cGOM* tool achieves a TPR of 79% and a TNR of 85%. For the AOI *bottle*, the TPR of the *cGOM* tool is 58% and the TNR is 98%. Table 1 shows the overview statistic of the fixation mapping both for SGM and for cGOM, including the number of fixations mapped [-], the average fixation duration [ms] and the standard deviation of the fixation durations [ms] for the two examined objects syringe and bottle.

*Efficiency of the manual mapping.* The analysts needed a total of 128 minutes on average for the mapping, with a standard derivation of 14 minutes. The subtasks were preparation, mapping and export of the data sample. The main time was needed to map all 4356 fixations. The exact sub-times for the total mapping process are presented in Table 2.

*Efficiency of the computational mapping.* The whole process using the algorithm required 236 minutes in total. The exact times of the subtasks of the mapping process are shown in Table 3. The mapping aided by the algorithm requires manual and computational steps. The *cGOM* tool spends most of the time for the steps of training and mapping that are solely conducted by a computer. The steps for collecting training images, manual labelling of the training images and export of the main study gaze data have to be performed by an operator. The labelled training set of this study included 72 images showing in total 264 representations of the object *syringe* and 32 representations of the object *bottle*. These three manual steps required 129 minutes in total.

**Figure 5:**
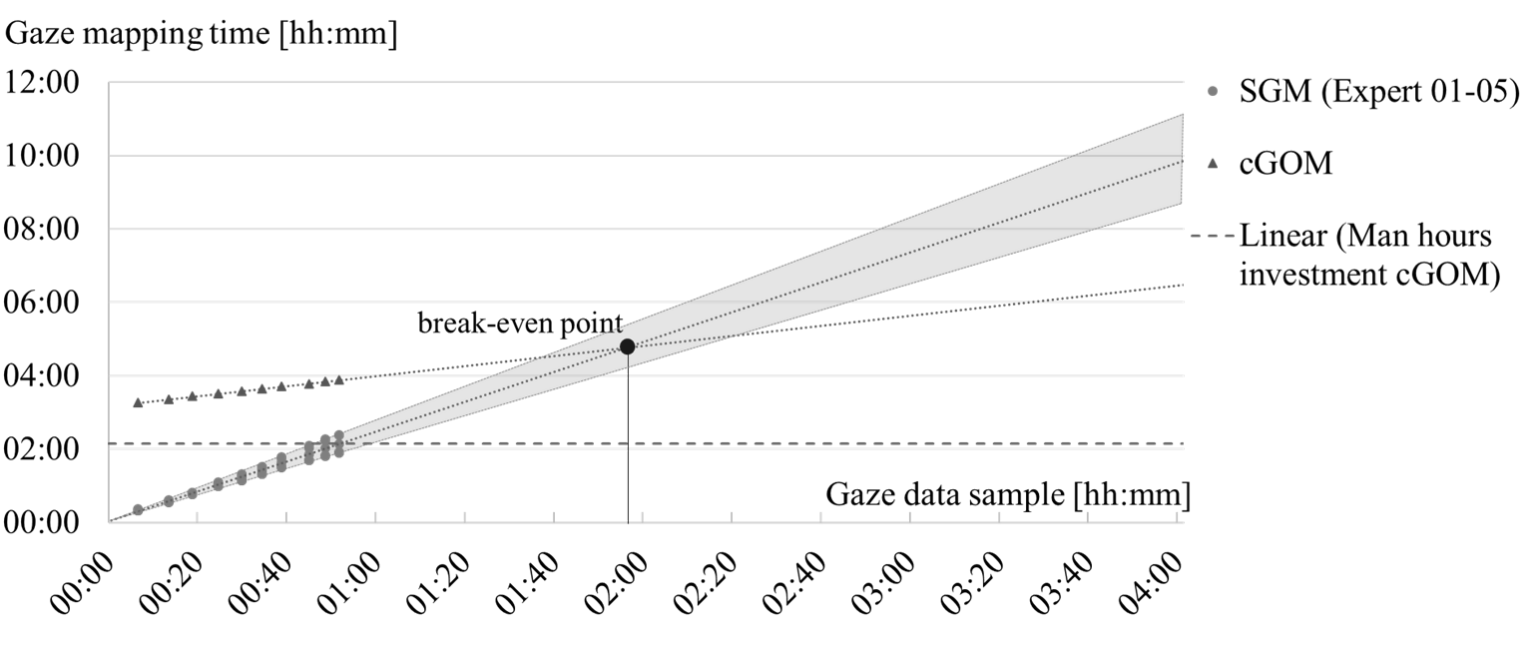
Graphical representation of data samples size in hours and minutes (abscissa) and time required for mapping in hours (ordinate) for manual mapping using SGM (●) and computational mapping using cGOM (▲). The linear approximation is based on the measured sub-times data. According to the approximation, a sample size of 4 hours video time would require 6.5 hours for the computational mapping and 10 hours for the manual mapping on average. The grey cone represents the distribution of the manual mapping and the linear continuation. The break-even point is at approx. 02:00h gaze data sample size. The break-even point for the man hours investment for cGOM is at approx. 01:00h gaze data sample size.

*Break-even point.* Figure 5 shows the approximated break-even point for the average time required for the manual mapping. The measurement points for the manual

mapping are the averaged interims for the single participant data sets. The completely analysed data sample (video time) totals up to 52 minutes. The start time for the data mapping was dependent on the required preparation for the manual and for the computational mapping. The average of the measured mapping times is extended by a linear trend line (dashed lines in Figure 5). The grey cone shows the extended linearized times of the fastest and slowest expert. All linearization is based on the assumption that the experts take sufficient breaks. The horizontal dashed line in Figure 5 indicates the time of the three manual steps required for the *cGOM* tool. For the manual *SGM* tool, the ratio between to-be-assigned gaze data sample and the required time for the mapping is 2.48 on average. For the *cGOM* tool, the ratio is 0.77 on average. The break-even point of the whole manual mapping procedure and the whole computational mapping procedure lies approximately at a data sample size of 02:00h. When comparing only the manual steps of both procedures, the break-even point reduces to a data sample size of approximately 01:00h (see Figure 5).

**Table 1:** Overview statistics of the fixations mapped by SGM and cGOM for the objects syringe and bottle. The applied statistics are number of fixation [-], fixation duration mean [ms] and fixation duration standard deviation [ms].

***Mapping characteristics***	SGM (syringe)	cGOM (syringe)	SGM (bottle)	cGOM (bottle)
Number of fixations [-]	2016	1934	79	82
Duration mean [ms]	445	434	311	248
Duration SD [ms]	568	571	296	146
				

**Table 2:** SGM - Overview of the mapping sub-times (mean and standard derivation in minutes) of the manual mapping by five professional analysts. The sub-tasks were preparation, mapping and export of the data sample.

***SGM (Semantic Gaze Mapping)***	Mean [min]	Standard derivation [min]
Preparation	1.5	1
Mapping	126	13
Export	1	0.5
**Total time**	**128.5**	**14**

**Table 3:** cGOM - Overview of mapping sub-times (mean and standard derivation in minutes) of the computational mapping by the algorithm. The mapping aided by the algorithm requires manual steps ^(#)^ and computational steps ^(*)^.

***cGOM (computational Gaze-Object Mapping)***	Mean [min]	Standard derivation [min]
Collecting training images ^(#)^	15	0
Manual labelling of the training images ^(#)^	113	38
Training of the neural network ^(*)^	67	0
Export of the main study gaze data ^(#)^	1	0.5
Mapping of the main study gaze data ^(*)^	40	0
**Total operator time**	**129**	**38**
**Total time**	**236**	**38**

## Discussion

The goal of the main study was to investigate whether the newly introduced machine learning-based algorithm *cGOM* offers the potential of replacing conventional, manual AOI evaluation in experimental setups with tangible objects. Therefore, manual gaze mapping using *SGM*, which was considered as ground truth, and *cGOM* were compared in regards to their performance. In the process, it was quantified whether the new algorithm is able to effectively map gaze data to AOIs (RQ1) and from which recording duration on the algorithm works more efficiently than the manual mapping (RQ2). Based on the results of this study we evaluate both research questions.

(RQ1) How effective is cGOM in assigning fixations to respective AOIs in comparison with the ground truth?

The two objects used during the handling task were deliberately selected because they represent potential challenges for machine learning. On the one hand, the syringes are partially transparent and constantly change their length during decanting. On the other hand, both the syringes and the bottle have partly tapered contours, which were assumed difficult to reproduce by close contour masks, in particular when working with small training data sets. According to the results presented in Figure 4, the assignment by the computational mapping has a TPR of 79% for the AOI *syringe* and 58% for the AOI *bottle*. For training the neural network, not only the number of training images but also the total number of object representations in these images is important. Since the stimuli consisted of five *syringes* and only one *bottle*, the 72 training images included 264 representations of the AOI *syringe* on which the neural network could learn, but only 32 representations of the AOI *bottle*. Due to the small learning basis, the masks produced by the algorithm sometimes did not include crucial features like the tip of the bottle (Figure 3).

For the AOI *syringe*, the TNR is slightly better than the TPR, whereas for the AOI *bottle* the TNR greatly exceeds the TPR. The relation between TPR and TNR can be well explained by the quality of the created masks. The masks tend to rather fill too little of the object than too much. The more the masks are directed inwards from the outer contour or exclude crucial features like in case of the bottle, the less true positives are registered, but the higher the probability of true negatives being recorded.

In line with the results, it can be concluded that for the AOI *syringe* the conformance with the ground truth is already promising, but can still be further increased. For the AOI *bottle,* there is no sufficient true positive rate yet, but with almost 60%, it is surprisingly high given the fact that the neural network was shown only 32 object representations of the *bottle* during training. In comparison, large online databases such as MS COCO work with hundreds to thousands of labelled images for training one single object. Objects already represented in the COCO data set may even be used in a plug-and-play approach without training, using only the gaze-object mapping feature of the presented algorithm. In contrast to the 72 images needed for the tailored approach of this study, the COCO dataset includes more than 320k labelled images in total (14). To improve the performance of the *cGOM* algorithm, the amount of training images and object representations can be increased until a sufficient TPR and TNR is reached.

(RQ2) At which recording duration does the efficiency of the computer-based evaluation exceed that of the manual evaluation?

The *cGOM* tool exceeds the manual evaluation when the respective procedure in total needs less time. The amount of data from which onwards the *cGOM* tool is faster than the manual evaluation is called the break-even point. For the break-even point one has to distinguish between the time for the total computational mapping procedure and the time, a person has to invest (see man hours investments of *cGOM* in Figure 5). The main part of the time is needed for training the algorithm on the training images, which is solely performed by the computer, and for the labelling of the training images, which has to be performed once by the analyst. For the total procedure and the consideration of the average speed for manual mapping by the experts, the break-even point lies at 2 hours of eye tracking recording. When focussing only on the time a person has to invest, this break-even point reduces to just 60 minutes of eye tracking recording.

After the preparation of the *cGOM* algorithm, the algorithm needs less than 8 minutes for every 10 minutes of eye tracking recording and thus is able to work in real time with a factor of 0.77. This is by far faster than the manual mapping, which has on average a ratio of 2.48 between recording length and time needed for the manual mapping. The difference in evaluation speed does not take into account symptoms of fatigue on the part of the analyst that increase considerably with longer evaluation times of *SGM*. With the given break-even points of only 2 hours of eye tracking recording or rather 1 hour, considering only the human working time, and the real-time capability of the mapping, the authors evaluate the efficiency of the *cGOM* tool exceeds the manual mapping for the majority of mobile eye tracking studies.

Due to the novelty of the algorithm presented in this study, there are several limitations regarding the results. First and foremost, to achieve the best possible results with a minimum amount of training images, the results presented are only valid in a laboratory environment with constant conditions. The amount of training data has to be higher to cover the possible variations in field studies. Further investigations are also required to determine which other objects are suitable for the presented algorithm and how the characteristics and number of objects influence the evaluation time. Although the comparison with SGM as a ground truth allows for a good assessment of the algorithm’s potential, it is questionable that the results of the manual mapping are always error-free due to the subjective evaluations by the analysts. The approach using five independent professional analysts tries to compensate this limitation.

Moreover, the gaze data set consisted of only 52 minutes video material and the derived linearization may not be true, as the human analysts cannot work uninterruptedly and the mapping speed decreases. The results for the measured times for the computational and the manual mapping may not be achieved when using a different computer system or mapping a different set of gaze data. The computational system used for this study has a state-of-the-art graphics processing unit (GPU), which is not comparable to the one of a standard computer regarding the speed and hence the time needed for training the algorithm and mapping the gaze data. Cloud computing, which was used in this study, lowers the burden as it continuously decreases the costs and democratises the access to the amount of computational performance needed.

As described in the introduction, AOI analysis of mobile, interactive eye tracking studies with tangible objects is still a time-consuming and challenging task. The presented *cGOM* algorithm is a first step to address this gap and complement state-of-the-art methods of automated AOI analysis (e.g. markers). For objects that are trained with a sufficient amount of training data like the AOI *syringe*, the algorithm already shows a promising TPR and TNR. Due to the early break-even point, both for the total procedure and in particular considering human working time, as well as the real-time capability of the mapping process, even hours of eye tracking recording can be evaluated. Currently, this would require an amount of time for the manual mapping that is not or difficult to realise, seen from an economical and a humane point of view. Consequently, this approach using machine learning for the mapping task promises to enable the mapping of a great amount of gaze data in a reliable, standardized way and within a short period. It lays the foundation for profound research on AOI metrics and reduces the obstacles many researchers still have when thinking about applying mobile eye tracking in their own research.

## Ethics and Conflict of Interest

The authors declare that the contents of the article are in agreement with the ethics described in http://biblio.unibe.ch/portale/elibrary/BOP/jemr/ethics.html. The ethics committee Zurich confirms that this research project does not fall within the scope of the Human Research Act (HRA) and therefore no authorization from the ethics committee is required. (BASEC No. Req-. 2018-00533, 27^th^ June 2018). All participants were asked to read and sign a consent form, which describes the type of recorded data and how this data will be used for publication. The authors declare to not have a conflict of interest regarding the publication of this paper.

## Acknowledgements

The authors Julian Wolf and Stephan Hess contributed equally to the publication of this paper and are both considered first author. We wish to thank all participants of this study for their time.
